# LLDNet: A Lightweight Lane Detection Approach for Autonomous Cars Using Deep Learning

**DOI:** 10.3390/s22155595

**Published:** 2022-07-26

**Authors:** Md. Al-Masrur Khan, Md Foysal Haque, Kazi Rakib Hasan, Samah H. Alajmani, Mohammed Baz, Mehedi Masud, Abdullah-Al Nahid

**Affiliations:** 1 Department of ICT Integrated Ocean Smart Cities Engineering, Dong-A University, Busan 49315, Korea; almasrurkhan@donga.ac.kr; 2 Department of Electronic Engineering, Dong-A University, Busan 49315, Korea; tusnow1990@gmail.com; 3 Electronics and Communication Engineering Discipline, Khulna University, Khulna 9208, Bangladesh; rakib17.rh@gmail.com; 4 Department of Information Technology, College of Computer and Information Technology, Taif University, Taif 21994, Saudi Arabia; s.ajmani@tu.edu.sa; 5 Department of Computer Engineering, College of Computer and Information Technology, Taif University, Taif 21994, Saudi Arabia; mo.baz@tu.edu.sa; 6 Department of Computer Science, College of Computers and Information Technology, Taif University, Taif 21944, Saudi Arabia; mmasud@tu.edu.sa

**Keywords:** autonomous cars, convolutional neural network, deep learning, lane detection, semantic segmentation

## Abstract

Lane detection plays a vital role in making the idea of the autonomous car a reality. Traditional lane detection methods need extensive hand-crafted features and post-processing techniques, which make the models specific feature-oriented, and susceptible to instability for the variations on road scenes. In recent years, Deep Learning (DL) models, especially Convolutional Neural Network (CNN) models have been proposed and utilized to perform pixel-level lane segmentation. However, most of the methods focus on achieving high accuracy while considering structured roads and good weather conditions and do not put emphasis on testing their models on defected roads, especially ones with blurry lane lines, no lane lines, and cracked pavements, which are predominant in the real world. Moreover, many of these CNN-based models have complex structures and require high-end systems to operate, which makes them quite unsuitable for being implemented in embedded devices. Considering these shortcomings, in this paper, we have introduced a novel CNN model named LLDNet based on an encoder–decoder architecture that is lightweight and has been tested in adverse weather as well as road conditions. A channel attention and spatial attention module are integrated into the designed architecture to refine the feature maps for achieving outstanding results with a lower number of parameters. We have used a hybrid dataset to train our model, which was created by combining two separate datasets, and have compared the model with a few state-of-the-art encoder–decoder architectures. Numerical results on the utilized dataset show that our model surpasses the compared methods in terms of dice coefficient, IoU, and the size of the models. Moreover, we carried out extensive experiments on the videos of different roads in Bangladesh. The visualization results exhibit that our model can detect the lanes accurately in both structured and defected roads and adverse weather conditions. Experimental results elicit that our designed method is capable of detecting lanes accurately and is ready for practical implementation.

## 1. Introduction

Road accidents have become one of the most common causes of death globally. Reasons such as reckless driving or even poor design and structure of the road have made road accidents more frequent. According to the World Health Organization (WHO), approximately 1.35 million accidental deaths occur annually worldwide [1]. Statistics show that traffic accident fatalities in developing and under-developed countries such as Bangladesh are 10 to 60 times more than in developed countries [2]. For example, the number of reported fatalities due to road accidents was 2376 in Bangladesh [1]. WHO estimated the actual number of fatalities to be as high as 21,316 [1]. From an economic standpoint, the cost of road accidents is estimated to be more than USD 4 billion, which is 1.3% of the total national GDP of Bangladesh [3]. Usage of autonomous vehicles instead of manual ones can reduce this rate by a large amount. The study shows that the hands-on implementation of an autonomous electric vehicle can be researched using a budget of around EUR 17,900 [4]. Thus, autonomous cars can be an important scheme in preventing road accidents. Autonomous cars often combine various modules, including adaptive cruise control (ACC), lane detection, driver awareness monitoring, etc. Among the different modules, lane detection is a vital aspect as it is used to develop lane-keeping assistance systems and lane departure warning systems. Literature shows that computer vision (CV) techniques have gained popularity among researchers for detecting lanes due to the availability of onboard visual sensors. Many methods are available for lane detection, both with and without including DL algorithms. Conventional feature-based methods that were applied in the earlier days of lane detection include different methods and algorithms such as the Canny algorithm [5] and the Hough Transformation method [6] to detect lanes. However, these methods fail to deliver a satisfactory performance when dealing with difficult road curvature, blurred lane lines, complex road patterns, etc. [7,8]. Researchers also applied model-based methods such as dynamic programming [9], Support Vector Machine (SVM) [10], B-Snake model [11], etc. These model-based methods leverage geometric parameters such as linear lines, quadratic curves, or cubic curves for lane detection. Though they have solved the problems partially, their performance is still not satisfactory for detecting lanes. Moreover, these methods need manual feature extraction, so these algorithms are faced with high computational and time complexity.

Among several DL methods, in recent years, CNN has been widely used in CV problems because of its ability to accurately perform image classification and segmentation tasks. CNN models do not require hand-crafted features; they can automatically extract meaningful features from input data. Several research papers have already focused on utilizing the above-mentioned CNN properties to learn robust feature representation and detect lanes even in complex scenarios. Though numerous methods have been demonstrated to be successful in lane estimation [12,13], it is challenging to deploy these methods in real-life applications. These methods only focus on delivering higher accuracy while detecting the lanes in the simulation environment. However, the system must be lightweight and capable of providing useful information with low render time to make it possible for real-life applications. Keeping this in mind, researchers have shown interest in developing different lightweight CNN methods for detecting lanes in real-time. In [14], Hou et al. proposed “E-Net”, an encoder–decoder structure-based model. The design of the encoder-decoder structure incorporated self-attention distillation and dilated convolution to make the model lightweight and achieve a lower render time. In another work [15], Lee et al. presented “DSUNet,” a novel fast, lightweight design for lane detection.

Despite the advantages, most CNN methods are trained with the data of good road conditions, i.e., roads with good pavements and roads without cracks. To date, researchers have not considered terrible road conditions that may severely affect the model’s performance and the autonomous vehicle’s performance. However, most developing countries, such as Bangladesh, lack proper infrastructure planning. The regular maintenance of communication pathways and the development of perfect road networks for deploying existing lane detection networks to introduce autonomous vehicles in these countries is tough to achieve within a short time due to financial limitations. So, this has become a conundrum for low-income countries, as very few related studies focus on solving these issues. Considering this colossal research scope, in this paper, we go beyond the limitations mentioned above and propose a lightweight CNN model named LLDNet, which can estimate lanes in both structured and defected road conditions as well as in adverse conditions. The following are the primary contributions of this research study:

- Developing a lightweight, accurate CNN model for lane detection.
- Assessing the numerical results of the proposed model and comparing them with other state-of-the-art methods.
- Testing the model’s performance in real-life scenarios considering Bangladesh’s structured and defected roads.

The remainder of this research is arranged as follows: The existing lane detection techniques are briefly discussed in Section 2. Section 3 provides a concise overview of the methodology of this work. Section 4 shows and discusses the experimental procedure and evaluation results of lane detection in various scenarios. Finally, Section 5 brings the paper to a conclusion.

## 2. Literature Review

Researchers from all over the world are devoting their efforts to developing methods for lane detection on roads. As a result, many methodologies have already been published in technical papers. Literature shows that computer vision-based methods have been widely utilized to solve lane detection tasks. These machine vision-based methods can be generally categorized into traditional and DL-based methods.

### 2.1. Traditional Methods

In the traditional methods, some basic features such as texture, gradient, geometric patterns, and colors are used to detect and fit lane lines on the road images. Aly et al. utilized the top view of the road images to extract necessary features using a Gaussian filter and employed the RANSAC algorithm to fit the lane lines [16]. Kamble et al. employed a canny edge detection algorithm to detect the boundary of the roads and expurgated the boundaries by Hough transformation [17]. Wennan et al. considered complex road environments, including colored lane lines, and traffic marking on the road, and successfully detected lanes under these conditions using a hyperbola model [18]. Hu et al. employed the Dynamic Region of Interest (DROI) technique for lane detection. The authors extracted the DROI points using interpolation [19]. Gao et al. proposed a fast and real-time lane detection framework based on the Gabor filter for detecting lanes on structured roads with multi-lanes and multi-scenes [20]. Wei et al. replaced the Canny edge detector with Robert operator and fused it to the hough transformation for improving the real-time performance of lane detection [21]. Andrei et al. improved the run time of an existing lane detection method by around 30%. They replaced the original hough transformation using the probabilistic transformation. They also used the parallelogram ROI instead of the trapezoidal one [22]. Wang et al. proposed a lane detection method based on the Catmull–Rom Spline, which can detect lanes in noisy environments by extracting the lane structures using control points [23]. Guotian et al. considered the problems of illumination changes and shadow effects during lane detection in real-time. The authors developed a top-hat transformation-based method to strain out the interruption of non-lane objects. They also utilized hough transformation and Kalman filter for lane fitting and correcting lane lines. Experimental results showed that their method was 95.63% accurate [24]. Li et al. considered the problem of broken boundary lines during lane detection tasks. They proposed a novel approach named parallel-snake (extension of active contour model) for solving this problem [25]. Yoo et al. proposed a vanishing point estimation method based on a probabilistic voting procedure for detecting lanes in complex road environments [26]. Though these methods can detect lanes in good condition, their performance is unsatisfactory in various scenarios, including curved roads, rainy days, changing illumination conditions, blurry lane lines, and others [7,8].

### 2.2. Deep Learning-Based Methods

Recently, among the DL models, CNN methods have shown mesmerizing performance in various computer vision tasks. The advantages of the CNN methods over the traditional methods are that they do not need hand-crafted features, and that the methods can extract the necessary features automatically and extract both local and global features for solving computer vision problems. Due to their advantages, CNN methods have already been adopted to solve the lane detection problems in numerous papers, which have pulled lane detection research to a new stage.

Kim et al. developed an end-to-end ego lane detection method by leveraging a transfer learning framework trained on the ImageNet dataset. The method was developed based on SegNet architecture and successfully detected the lanes without any post-processing [27]. In [28], SegNet was also used as the backbone network for developing a lane detection method named LaneNet. LaneNet considered the lane detection task as an instance segmentation to detect multiple lanes and handle the issue of lane changes. Bruls et al. utilized a conditional random field (CRF)-based method to create a label in a weakly-supervised way from multimodal sensor data instead of manually annotating the data. These images were later used in a U-shaped network for accurate lane detection [29]. Khan et al. employed a fully convolutional network (FCN) architecture incorporating a secondary layer protection scheme for detecting the lanes as well as ensuring their prototype vehicle is always kept inside the track [30]. Chng et al. improved a lane detection method named RONELD by making it more robust to detect lane changes. Their advanced method named RONELDV2 detected lane point variance for finding more accurate lane parameters, which accelerated their method’s performance [31]. Lee et al. proposed a novel lightweight lane detection method named DSUNet for detecting the lanes in real-time. The model outperformed the original U-net model in terms of both model size and inference time [15].

Furthermore, after implementing the model on the actual car, it outperformed the U-net model in terms of lateral error while navigating on a real road [15]. Zhang et al. introduced a novel method named RS-Lane for detecting multiple lanes in challenging scenarios. The method was developed based on LaneNet, and the split attention concept of the RSNet was also employed [12]. Zou et al. utilized multiple frames from a continuous driving scene instead of a single frame to predict the lanes on the road. The proposed method was a hybrid architecture, i.e., the combination of CNN and RNN. The CNN block extracted features from multiple images and made time-series data, while the RNN block predicted the lanes using the passed time-series data [13]. Chng et al. developed a method for predicting the lane marking using the point cloud data extracted from light detection and ranging (LiDAR). The proposed system can also estimate the lane width as well as can report the location of the lane marking gaps [32]. Li et al. developed a novel method named ZF-VPGNet for detecting lanes on the road. The model was developed for multi-task learning. Another version of the method is CZF-VPGNet which can be easily implemented in the embedded devices without affecting accuracy [33]. Chen et al. designed a Spatio-temporal attention module (STAM) to integrate into a VGG-16-based FCN network for predicting lanes from multiple consecutive input frames. The method solved the problems of occluded lanes by using the history of the previous frames [34]. Lee et al. presented a multi-task deep learning architecture for detecting potential drivable areas and lane line segmentation. The MobileNetV2 architecture was used as the backbone of the encoder part, while three decoder sections were utilized for the multi-task learning [35]. Perng et al. proposed a hybrid model by combining an autoencoder named CAE and a hyperbolic model for detecting lanes in structured and unstructured roads. The models extracted the feature points of the lane using the CAE and later fit the lane lines by utilizing the hyperbolic model. The model was implemented on an Nvidia Jetson Tx and tested on three different scenarios [36]. Tabeleni et al. proposed a novel lightweight deep learning network named PolyNet based on the deep polynomial regression for detecting lanes. The proposed model is not only accurate but also efficient enough to maintain 115 FPS in TuSimple dataset [37]. Munir et al. utilized an event-based vision sensor instead of the RGB camera to solve illumination variance and motion blur. They developed a deep learning model named LDNet comprising an encoder, ASPP block, and an attention-guided decoder block. The model utilized the images from the event-based vision sensor and can detect the lanes accurately [38]. In the most recent years, Tabelini et al. proposed a CNN model named LaneATT for detecting lanes with more efficacy and efficiency. The proposed model considered global information as crucial and used an anchor-based attention module for detecting lanes in case of occlusion and missing lane markings. The authors claimed that their model achieved FPS up to 250 [39]. Ko et al. developed a lightweight CNN model named point Instance Network (PINet) for handling the problems of multiple lines and limitation of computing power during detecting lanes in real-time. The proposed model contains several stacked hourglass networks where the number of the hourglass networks can be clipped based on the power of the onboard device [40]. Haris et al. considered the necessity of including a decoder module which is ignored in many existing methods for predicting lanes in real-time. Consequently, the authors designed a novel framework based on object feature distillation which can be applied to any CNN-based lane segmentation network and can produce promising results with no additional cost [41]. In another work, Haris et al. proposed a CNN model for handling the issue of the narrow line at the vanishing point of lanes. The authors designed a lane detection network, as well as a lane offset estimation algorithm in the decoder part of their model for improving the model’s performance [42]. Li et al. utilized the Mask R-CNN model for detecting lanes using the point-cloud data from a 3D LiDAR. For reducing the computational power the authors aggregate the point cloud data into a 2D-image space before using the affine transformation. Experimental results displayed their model’s efficacy during detecting lanes [43]. Haris et al. proposed an asymmetric kernel convolution (AK-CNN) for detecting lanes under complex traffic conditions. The authors included a weight-sharing function on the CNN to make the model lightweight. Consequently, their method achieved FPS up to 84.5 [44].

The literature shows that CNN methods have already been improved for detecting lanes in numerous scenarios. However, very few research works have focused on developing lightweight CNN methods that can estimate lanes accurately in adverse weather conditions and on bad and unstructured roads due to the lack of training data on roads of poor quality. So, in this study, we have developed a lightweight CNN model which is trained on a mixed dataset (one of the datasets, named Cracks and Potholes in Road Images Dataset [45], contains images with cracked pavements) for detecting lanes and tested them in different weather and road conditions.

## 3. Proposed Model

**Figure 1:**
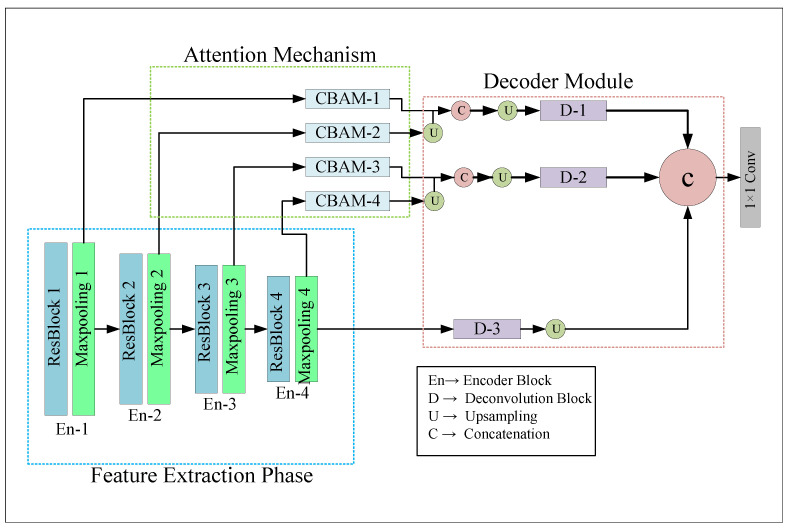
Structure of the Proposed LLDNet architecture. Our code is available at https://github.com/Masrur02/LLDNet (accessed on 21 June 2022).

Lane detection can be considered a semantic which classifies “Lane” and “Non Lane” pixels into two groups. In this work, we proposed attention-based encoder-decoder architecture, namely LLDNet, for detecting road lanes in real-time. The Deep Learning model was trained to preserve the necessary spatial information for good prediction. The overall structure of our proposed LLDNet is illustrated in Figure 1.

As shown in Figure 1, the framework of the LLDNet was composed of three parts, including the feature extraction phase, Convolutional Attention Block Module (CBAM) [46] part, and the Decoder part. The feature extraction phase or the encoder branch of our model extracts the necessary features and generates low-level to high-level feature maps from the RGB images of size 80 × 160 pixels. Later, the features generated from the encoder part are passed through the attention module block for obtaining the advanced features. The primary purpose of the attention mechanism in our model is to focus more on the road portion of the images and ignore the other objects in the images (e.g., sky, trees, pedestrians, and others), which will accelerate the model’s performance and save processing time. Finally, the decoder part of our model reconstructs the feature maps extracted from both the encoder and the attention module to produce the predicted images with the exact resolution of input images. The design of each branch of our model is discussed briefly in the following subsections.

### 3.1. Encoder Module

**Figure 2:**
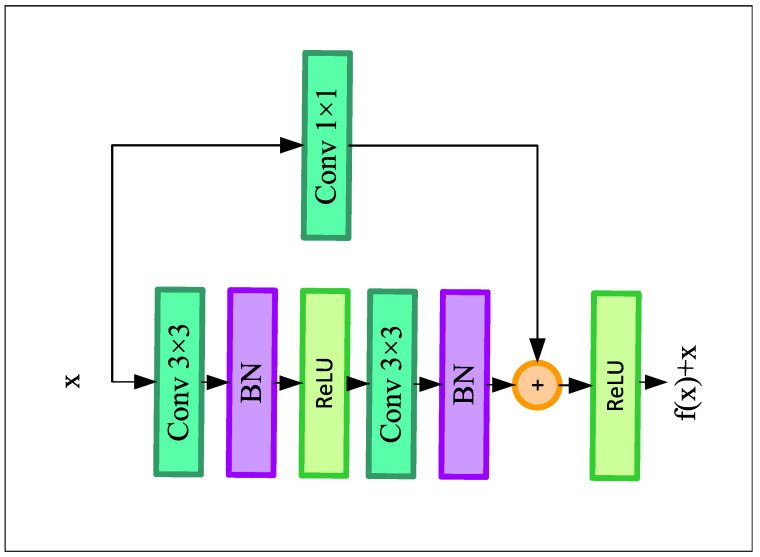
Structure of the residual block.

The encoder module of our network composes of four residual blocks, each of which is followed by a 2 × 2 max-pooling layer. The residual blocks help to build a deeper convolutional neural network without having the issue of vanishing gradient problems. Furthermore, it enhances the channel inter-dependencies while also alleviating the computational cost of the model. Figure 2 demonstrates the architecture of the residual blocks.

Each residual block consists of two convolution blocks, where the convolutional blocks consist of a 3 × 3 convolutional layer with the same number of filters (from 8 to 64) followed by a Rectified Linear Unit (ReLU) activation function and a batch normalization layer. A skip connection followed by a 1 × 1 convolution layer has been employed, which adds the input of the residual block directly before the last ReLU layer. This skip connection helps the deep learning model for solving the vanishing gradient problem by adding the feature map of the *l*th layer to the (l+2)th layer of the model. Finally, the output of the resblock goes through the max-pooling layer and downsamples the input feature map by two times. The first encoder block of our LLDNet gets the input of size H × W × C. Here, C is the input channel that equals 3 for RGB images. So, after the first encoder block, the output feature size becomes H/2 × W/2 × C. Here, C equals the number of filters used in the corresponding encoder block. Finally, at the last encoder block, we obtain the feature map of size H/16 × W/16 × C.

### 3.2. Attention Mechanism

**Figure 3:**
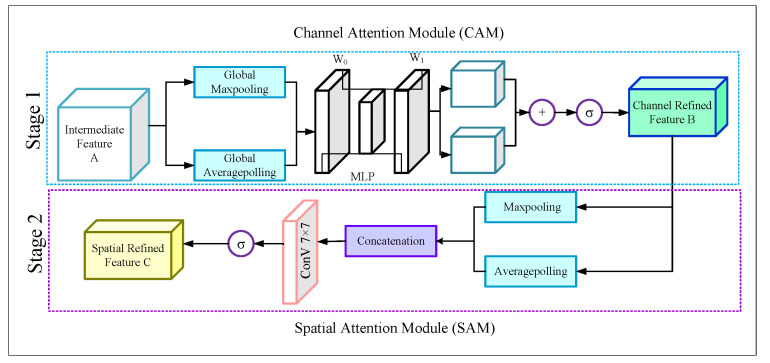
Structure of the CBAM module.

From the different stages of the encoder module we obtained activation maps of size 40 × 80 × 8, 20 × 40 × 16, 10 × 20 × 32, and 5 × 10 × 64, respectively. Later, we passed these activation maps through four (one for each activation map) Convolutional Block Attention modules (CBAMs) [46] in order to obtain the advanced features. The structure of the CBAM blocks is illustrated in Figure 3.

The figure displays that each CBAM block is incorporated with the Channel Attention Module (*CAM*) and the Spatial Attention Module (*SAM*). The CBAM module finds the important channels from the intermediate feature map A by applying a 1D CAM *F*${}_{CAM}$∈$R^{C\times 1\times 1}$ and produces a channel refined feature map *B*. Mathematically,

(1) $$B=F_{CAM}\left(A\right)⊗A$$

Later the CBAM module applies a 2D SAM, *F*${}_{SAM}$∈$R^{1\times H\times W}$ on the feature map *B* and finally generates a spatial attention map.

(2) $$C=F_{SAM}\left(B\right)⊗B$$

#### 3.2.1. Channel Attention Module

In the CAM, the intermediate feature map *A* goes through a Global average pooling *g*${}_{a_{p}}$ and Global maxpooling *g*${}_{m_{p}}$ layer. Thus the CAM generates two descriptor features, *P*${}_{ap}$, *P*${}_{mp}$. Mathematically,

(3) $$\begin{array}{c} P_{ap}=g_{a_{p}}\left(A\right)\\ P_{mp}=g_{m_{p}}\left(A\right) \end{array}$$

Later, both the *P*${}_{ap}$ and *P*${}_{mp}$ produce output features by sharing a multi-layer perceptron (*MLP*). After that, the outputs were added by using an element-wise summation, and finally, the merged output passed through a sigmoid (σ) for generating the output feature *F*${}_{CAM}$(*A*). Mathematically,

(4) $$F_{CAM}\left(A\right)=\sigma \left\{MLP\left[P_{ap}\right]⊗MLP\left[P_{mp}\right]\right\}$$

For the MLP, the number of hidden layers is selected as $R^{C/r\times 1\times 1}$ to reduce the computational cost, where *r* is the reduction ratio. If the shared weights of *P${}_{ap}$* and *P${}_{mp}$* are *w*${}_{0}$∈$R^{C/r\times C}$, *w*${}_{1}$∈$R^{C\times C/r}$ then Equation (5) can be expressed as,

(5) $$F_{CAM}\left(A\right)=\sigma \left\{w_{1}\left[w_{0}\left(P_{ap}\right)\right]⊗w_{1}\left[w_{0}\left(P_{mp}\right)\right]\right\}$$

Now according to Equation (1), this *F*${}_{CAM}$(*A*) will be element-wise multiplied by the intermediate feature *A* and produce a channel refined feature *B*, which will be the input of the SAM.

#### 3.2.2. Spatial Attention Module

The SAM takes the output o the CAM and sends the feature map *B* through a maxpooling *m*${}_{p}$ and an average pooling *a*${}_{p}$ layer, and we obtain

(6) $$\begin{array}{c} Q_{ap}=a_{p}\left(B\right)\\ Q_{mp}=m_{p}\left(B\right) \end{array}$$

Then both of the two dimensional outputs *Q*${}_{ap}$∈$R^{1\times H\times W}$ and *Q*${}_{mp}$∈$R^{1\times H\times W}$ were concatenated as

(7) $$Q=(Q_{ap},Q_{mp})$$

Later, the concatenated activation map *Q* is passed through the 7 × 7 convolutional layer. Then the map is followed by a sigmoid function (σ) to produce the output feature $F_{SAM}\left(B\right)$. Mathematically,

(8) $$F_{SAM}\left(B\right)=\sigma \left\{conv^{7\times 7}\left[Q\right]\right\}$$

Finally, according to Equation (2), this $F_{SAM}\left(B\right)$ will be element-wise multiplied by the channel-refined feature *B* and produce a spatial refined feature *C*, which is the final output of the CBAM block.

So, by using the CBAM blocks after the encoder stages, we obtained four refined activation maps that focus more on the road portions of the images. The size of the refined activation maps is the same as the intermediate activation maps extracted from the encoder stages.

### 3.3. Decoder Module

In the decoder module, first, we take the outputs of the blocks named CBAM-1, CBAM-2, CBAM-3, CBAM-4, and pool4. The size of the feature maps of these blocks are 40 × 80 × 8, 20 × 40 × 16, 10 × 20 × 32, and 5 × 10 × 64, respectively. Later we produced two activation maps named C1 and C2 by concatenating CBAM-1 & CBAM-2 and CBAM-3 & CBAM-3. Concatenation of all the activation maps together from the attention blocks may lose some necessary features, so we merged them pair-by-pair for combining the low-level features (CBAM-1 & CBAM-2) and high-level features (CBAM-3 & CBAM-4). However, before concatenating, we conducted 2 × 2 upsampling both on the CBAM-2 and CBAM-4 for converting them in the same shape as CBAM-1 and CBAM-3. After that, we utilized three parallel deconvolutional blocks (D-1, D-2, D-3). The inputs of these three deconvolutional blocks are CBAM-1, CBAM-2, and Maxpooling 4. The purpose of using the activation map of pool4 is that this activation map contains the original features extracted by all the encoder blocks. However, we applied 2 × 2, 4 × 4, 16 × 16 upsampling to the inputs of the deconvolutional blocks, respectively, for converting them into the same shape as the input images. The deconvolutional blocks consist of two 3 × 3 conv2Dtranspose layers [47] with the same number of filters from (32 to 8), followed by a batch normalization layer. After the deconvolutional blocks we obtain three activation functions with the shape of 80 × 160 × 32, 80 × 160 × 16, and 80 × 160 × 8. Later, we concatenate all the three feature maps to combine all features (low level, high level, refined) in a single map. After the concatenation, the shape of the feature map becomes 80 × 160 × 56. Finally, we utilized a 1 × 1 convolution with one filter and obtained the predicted image with shape 80 × 160 × 1.

## 4. Experiments and Results

In this section, we carried out a few experiments to evaluate the outcomes and robustness of the proposed model. We evaluated our model’s performance by considering several numerical results and predicting lanes from road images in various scenarios.

### 4.1. Dataset

In this work, we constructed a mixed dataset by combining the “Udacity Machine Learning Nanodegree Project Dataset” [48] and the “Cracks and Potholes in Road Images Dataset” [45]. The first dataset was collected by a smartphone and contained 12,764 training images. The images were extracted from 12 different videos filmed at 30 fps. Though the images’ original size was 1280 × 780 pixels, the images were later resized to 80 × 160 pixels. For labeling the images, the images were first calibrated using OpenCV for correcting the camera’s inherent distortion. Later, the perspective transformation was used to put the road lines on a flat plane. Finally, this process produced ground truth images where the white pixels denote the lane areas and the black pixels denote the non-lane areas. The original images and the labeled images were then converted into two different pickle files and uploaded to Mr. Michael Vigro’s GitHub page [48]. After downloading the pickle files, this work converted them into NumPy files. The speciality of this dataset is that it contains images of different weather conditions, different road curvatures, and different lighting conditions. The other dataset of our work collected images by using a Highway Diagnostic Vehicle (DHV). This dataset contains 2235 images which were extracted from a few videos filmed at 30 fps with a resolution of 1280 × 729 pixels. The speciality of this dataset is that most of the images in this dataset contain images with cracks and holes on the road. However, the roads on the images are not heavily damaged; rather, the images contain roads with minor damage. Each image of this dataset contains three mask images, including lane marking, hole marking, and crack marking masks. However, we only consider the lane marking masks images of this dataset. The mask images were produced in a way where the white pixels denote the lane areas and the black pixels denote the non-lane areas. The original images of this dataset were uploaded in jpg format and the labeled images were uploaded in png format. The primary purpose of mixing these two datasets in our work is that we want to develop a robust system. The system can detect the lanes in adverse weather or lighting conditions, even if the roads are defective and unstructured. We resized the second dataset in the same size as the first dataset for mixing the two datasets. After that, the original and labeled images were imported as NumPy arrays and stored in two different Numpy files. And these two NumPy files were merged with the Numpy files of the previous dataset for generating a mixed dataset. The mixed dataset utilized in this work can be found https://drive.google.com/file/d/1S23Ac0_hbOktV0rE2q0IkQWpQjUfkMTB/view?usp=sharing and https://drive.google.com/file/d/1I264WVBL3Dyp_4PTfEYkVIDkg_Yn5gJJ/view?usp=sharing (Accessed on 21 June 2022). Finally, the mixed dataset contains 14999 images where the shapes of the original images are 80 × 160 × 3, and the shape of the labels are 80 × 160 × 1. Later, we split the entire dataset into a 7:3 ratio using the train _test _split function of the scikit-learn library to separate the training and test sets.

### 4.2. Implementation Details

For USA or Canadian companies, please provide the company, city, abbreviated state name, USA/Canada). Please check and confirm throughout the paper. We used python version 3.6.13 as the development language and Keras version 2.6.0 as the Deep Learning framework. In the training of our model, we used Adam optimizer, dice coefficient loss function, a batch size of 64, the learning rate of 1e${}^{-4}$, and the number of epochs was 100. We trained the model and conducted our experiments in a computer configured with Windows 10 operating system, 32 GB RAM, Intel core i9-11900k @ 3.50 GHz CPU processor, and NVIDIA Geforce RTX 3080Ti graphics card.

### 4.3. Performance & Robustness of the Model

#### 4.3.1. Quantitative Results

Verifying the performance of our model in terms of the mixed dataset and comparing it with other benchmark models is one of the ways to evaluate our model’s accuracy and robustness. As the target of our model is to classify the “Lane” and “Non-Lane” pixels, we employed a few metrics to appraise our model, including pixels’ accuracy, Dice-coefficient, and Intersection over Union (IOU). The pixel accuracy is considered as the percentage of correctly classified pixels in the case of a binary segmentation task. However, pixel accuracy is not the optimal criterion to assess the segmentation task because of the class imbalance problem. In the case of lane detection, the images in the dataset are highly imbalanced for the lion’s share of the Non-lane pixels. On the other hand, the Dice coefficient and the IOU are considered more effective metrics as they depend on the overlapping area between the ground truth image and the predicted image. The following mathematical equation can express the metrics.

(9) $$\begin{array}{c} \mathrm{Dice}\;\mathrm{Coefficient}=\frac{2\sum Y_{p}Y_{t}}{\sum Y_{p}+\sum Y_{t}}\\ \mathrm{IoU}=\frac{\sum Y_{p}Y_{t}}{\left(\sum Y_{p}+\sum Y_{t}\right)-\left(\sum Y_{p}Y_{g}\right)} \end{array}$$

**Figure 4:**
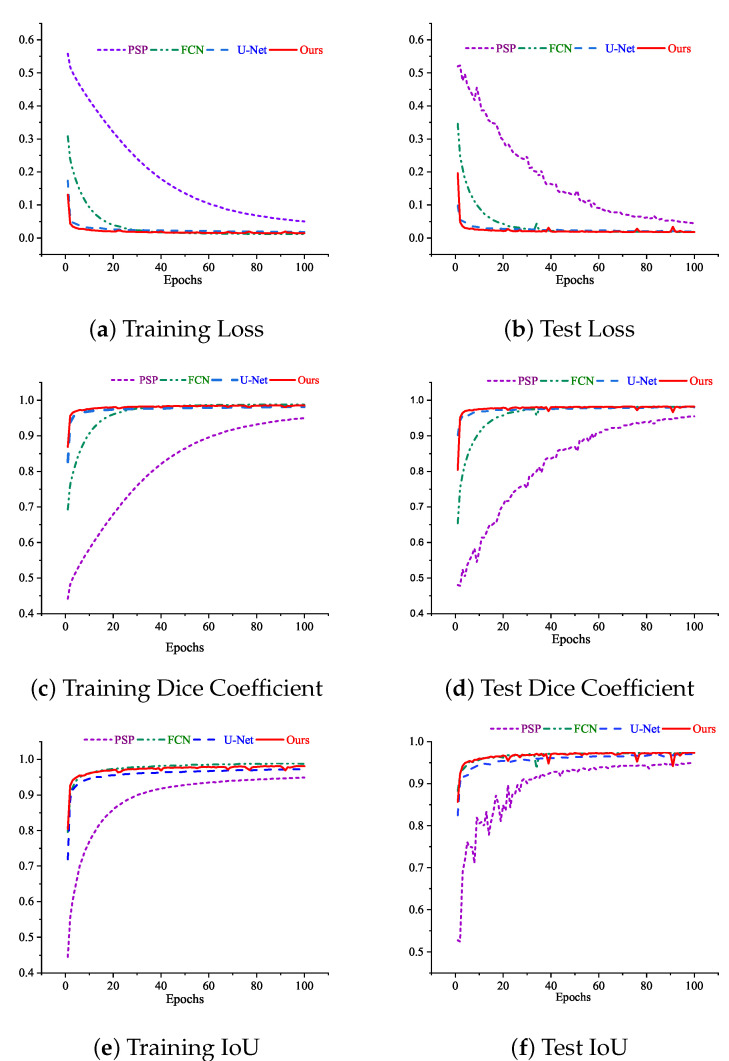
Curves of loss, dice coefficient, and IoU while training and testing the models at 100 epochs.

The equation shows that the Dice coefficient illustrates the two overlapping areas divided by the total of pixels. At the same time, the IOU represents the overlapping area divided by the union area between the ground truth and the predicted images. Figure 4 shows the loss, Dice coefficient, and IoU trend over the epochs of both training and test sets for our as well as other state-of-the-art models. The models we have chosen to compare with our work are PSPNet, FCN, and U-net respectively. We have chosen these models as benchmarks as they are popular as well as widely used CNN architectures for performing semantic segmentation tasks and show promising results in benchmark datasets [49]. It can be seen from Figure 4 that the test curves of the PSPNet were not good. The curves keep fluctuating abnormally throughout all the epochs. The numerical results started at a low level and did not reach even closer to the other models at the 100th epoch in terms of all metrics.

On the other hand, the FCN and the U-net model were trained well. There is no fluctuation on the curve throughout the training and test, and it becomes stable after a few epochs. Furthermore, there is not much difference between the training and test results, indicating that the models did not overfit or underfit. However, it is apparent from the picture that, though there is a little fluctuation in the test Dice coefficient and test IoU curve of our proposed model, our model outperformed the other models in terms of both Dice coefficient and IoU. The Dice coefficient and IoU curves of our model started rising rapidly from the first to around fifth epoch. After the 5th epoch, it raised slowly and started to become stable; but, around the 25th, 40th, 79th, and 95th, four little fluctuations of different degrees were experienced. However, our model was able to cope with this fluctuation, and from the very next epochs, the curves became stable again. Finally, our model obtained better results than the other models.

Table 1 presents the comparison of our developed model with the other models in the perspective of the previously mentioned metrics on the test set. Moreover, we also included the total number of parameters and the weight file size on the comparison table to better understand the model’s performance and robustness. According to Table 1, our developed model outsails all the other compared models in terms of all metrics. Let us analyze the Dice coefficient result, as the Dice coefficient is the most critical metric for the segmentation task. It can be seen from the table that our model obtained 2.77%, 0.16%, 0.05% Dice coefficient than the other models respectively. The segmentation metrics of the U-net, FCN and our proposed model do not differ significantly from each other. However, the total number of parameters and the size of our model’s weight file are significantly lower compared to those of the other models. The FCN and the U-net produced competitive results with our model. However, the number of parameters of these models is 5.26 times and 7.46 times greater, respectively, than our model, which indicates that our model needs lower computational complexity to obtain better results than the other models. Furthermore, the weight file size of our model is 8.70 times and 12.21 times smaller than the weight file of the FCN and U-net model, respectively. This indicates that our model will have better adaptivity and applicability on embedded devices to implement a real-life robotic vehicle that detects lanes in real-time. So, from Table 1, it can be concluded that our proposed model is lightweight and can produce more accurate results.

#### 4.3.2. Qualitative Results

In this subsection, we will present some visualization results obtained by our proposed model. To check our model’s robustness, we feed some videos of Bangladeshi roads to the model. The videos were collected from Youtube considering several conditions, and we predicted the lanes by our model. The experimental conditions we choose in our work for proving the robustness of our model include

- Perfect road with normal weather condition;
- Curvy road condition;
- Rainy condition;
- Night condition;
- Defected pavement and occluded lane line condition.

The location of the experimented scenarios, experimental setup, and the obtained results in each condition are presented below.

#### Perfect Road with Normal Weather Condition

**Figure 5:**
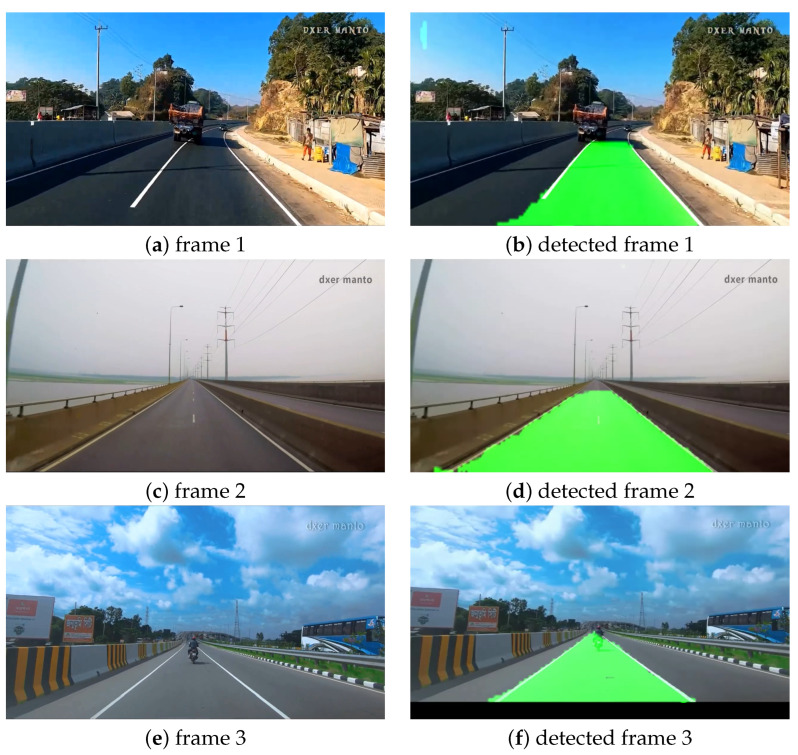
Visualization of lane detection in perfect road and weather conditions.

We have executed our first experiments on perfect road and weather conditions. For this experiment, we considered different highways in Bangladesh, including the Dhaka–Chittagong Highway, Mawa Express Highway, and the Jamuna Bridge (connecting bridge of the capital city and northern districts of Bangladesh). The lanes on these roads are well marked, and the pavement condition is also good. We considered perfect weather and illumination conditions as well for this experiment. The videos were collected from the Youtube channel of a Bangladeshi travel blogger who collected these videos while driving a car [50,51,52]. Figure 5a,c,e show the original frames collected from Dhaka–Chittagong road, Jamuna Bridge, and Mawa road, respectively, and Figure 5b,d,f illustrate the predicted images of the corresponding frames. From the figure, it can be seen that our model predicted the lanes correctly in almost all cases. However, if we look closely, the left lane of Figure 5b exceeded a tiny portion. This issue might result from the long break of the lane lines. Furthermore, one potential cause is that as the videos were collected randomly (without the intention of research), the camera angle may become distorted when very close to the lane line which finally result in such a situation.

#### Curvy Road Condition

**Figure 6:**
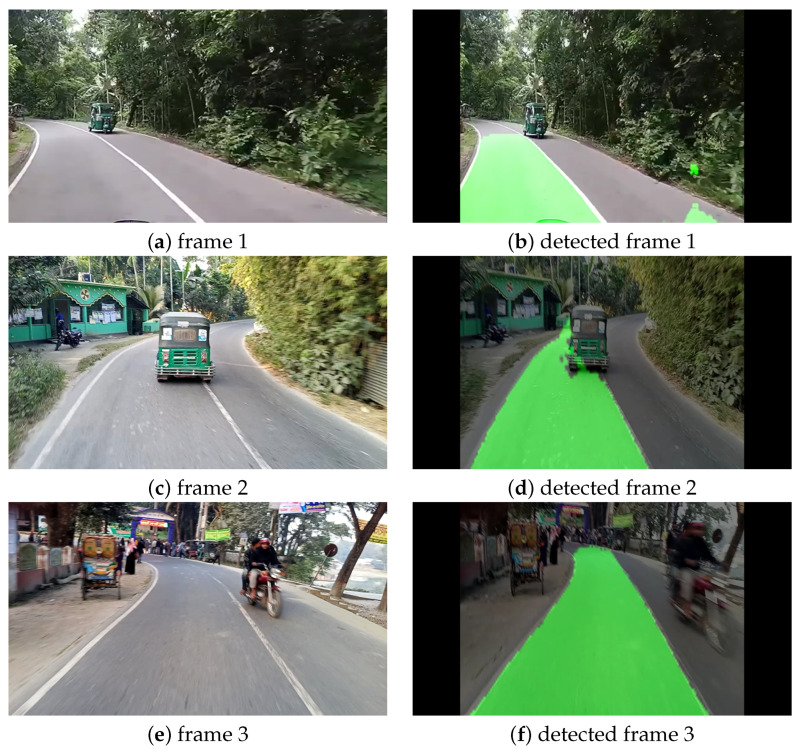
Visualization of lane detection on curvy road condition.

For our second experiment, we chose the curvy road condition, as it is one of the most challenging assignments for the CNN models to detect cracks accurately in a curvy road. In this case study, we collected videos by ourselves from a road named “Daulatpur –Tangail Rd” located in a city of Bangladesh named Tangail. The videos were collected by a Techno spark 7 smartphone with a resolution of 1920 × 1080 pixels at 30 FPS while traveling by motorcycle at approximately 30 km/h. Figure 6 illustrates the original frames and the corresponding predicted frames with lanes marked in green color at the different locations of the road mentioned above. From Figure 6, it can be observed that detected frame 2 and detected frame 3 are perfect. Even though in detected frame 2 there was another vehicle in front of our motorcycle, our model still detected the lane accurately. However, in detected frame 1, our model mistakenly predicted a tiny scattered portion of the road as a detected lane. As our model is a lightweight model with lesser parameters, we neglected this little mistake and considered this frame a good result.

#### Rainy Weather Condition

**Figure 7:**
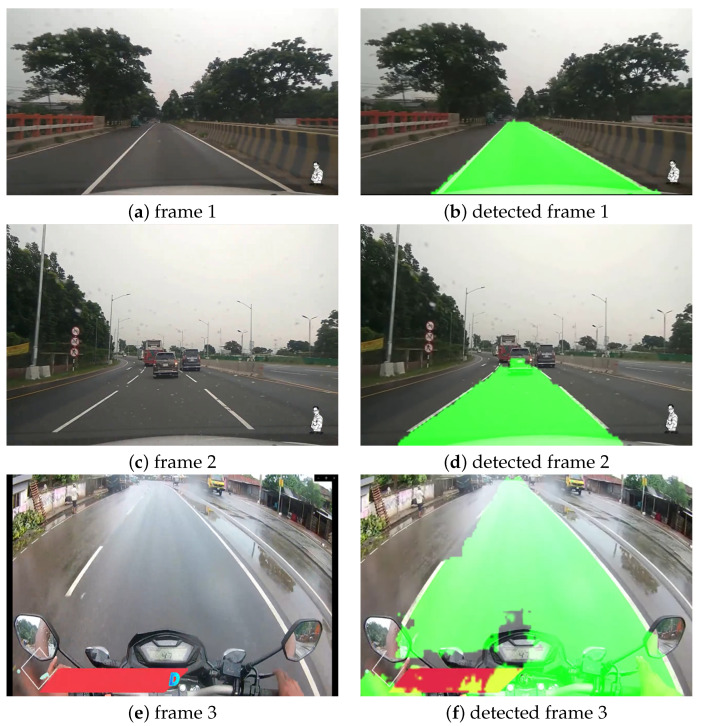
Visualization of lane detection in rainy weather condition.

We also considered rainy weather conditions as one of the experimental cases of our study. We considered the Dhaka–Chittagong Highway and the Magura district road, as the location of this experimental case study. For this experiment, we collected videos from Youtube [53,54], filmed during light and heavy rain. Figure 7 illustrates the original and predicted images in rainy weather conditions. In Figure 7 frame 1 and frame 2 are from Dhaka–Chittagong highway during light rain, and frame 3 is from Magura district road after heavy rain. From Figure 7, it can be seen that all of the frames were predicted accurately by our model. However, if we look closely, it can be seen that the lane prediction in detected frame 3 was a bit distorted at the bottom part of the image. The camera was set up on the biker’s helmet, and as it captured a portion of the bike, our model was a little confused. If the camera captured only the road portion, the entire image would be predicted well, just like the other portion of detected frame 3.

#### Night Condition

**Figure 8:**
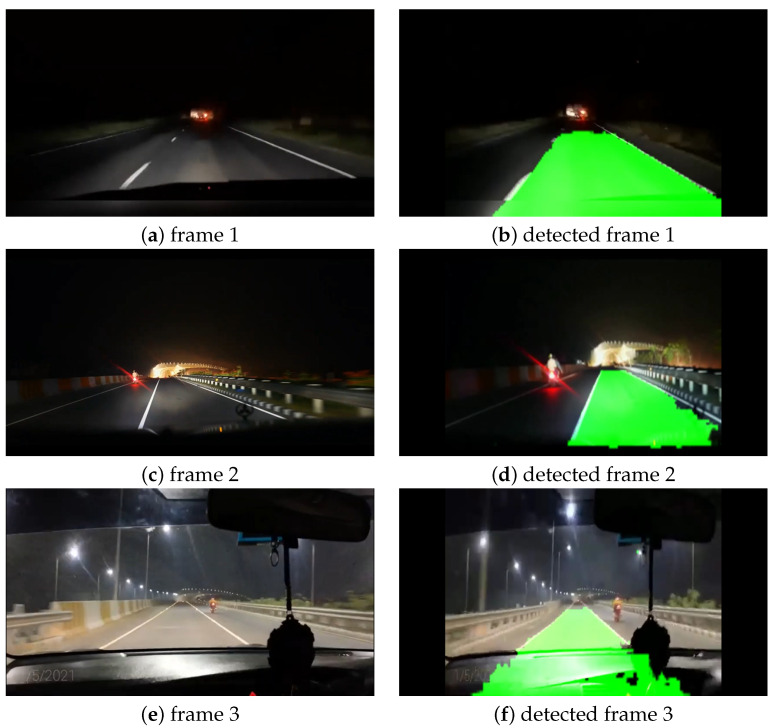
Visualization of lane detection on night condition.

CNN models may find it challenging to predict lanes in low-illumination conditions. So, for evaluating our model, we have chosen to use the night time condition when predicting lanes. Like the first and third experimental case studies, we also collected the videos from Youtube for this experiment [55,56,57], filmed at the Dhaka–Chittagong highway and Mawa expresses highway at night time. Figure 8 shows the original frames as well as the predicted frames at night time. Figure 8a is from the Dhaka–Chittagong highway, and the other two images are from the Mawa express highway. We can observe at Figure 8 that our model predicted the lanes accurately even at night time. However, in detected frame 3, our model predicted a portion of the car’s dashboard and the lane. This problem most likely occurred due to the adjacent continuous frames.

#### Defected Pavement and Occuladed Lane Line Condition

**Figure 9:**
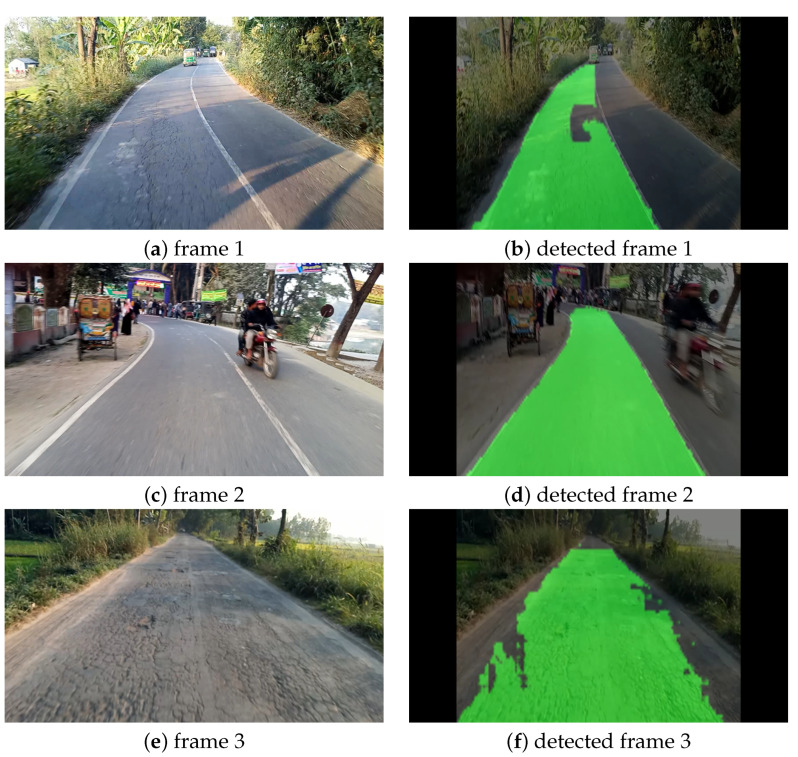
Visualization of lane detection on defected road condition.

In Bangladesh, there are many roads where the pavement condition is not good. Sometimes, there are many cracks and big holes on the road. Moreover, the lane marking is not clear; sometimes, it vanishes. So, in this experimental study, we have considered the scenario mentioned above and challenged our model to predict the lanes in such adverse scenarios. For this experiment, we collected videos from the Daulatpur–Tangail road located in Bangladesh in Tangail using the same setup described in the curvy road condition. Figure 9 displays the real and predicted frames by our model in defective pavement conditions. From frame 1, it can be seen that though there are many cracks on the pavement, our model still predicted the lane accurately except for a bit of discontinuity in the prediction. In the case of frame 2, it can be observed that our model predicted the lane boundary accurately despite the unclear and occluded right lane line. Finally, in frame 3, we have chosen a road with many cracks and holes in the pavement. Even the lane marking in the road has totally vanished. Despite all this adversity, our model accurately predicted the lane. Though there is an unpredicted portion on the edges of the image, the prediction does not exceed the road boundary, which is good enough for a vehicle to navigate safely even in this awful road condition.

## 5. Conclusions

In this paper, we have proposed a novel lightweight CNN model for accurate lane detection. With refined information extracted by the attention modules, our proposed method accomplished robust and reliable lane detection even in adverse conditions. The assessment showed that our method was capable of achieving state-of-the-art performance, with higher Dice scores and fewer parameters when compared state-of-the-art algorithms. Furthermore, the suggested method showed outstanding qualitative results in various difficulty settings, demonstrating its robustness. Though our method works on both structured and unstructured roads, the performance on unstructured roads suffers slightly when the road is extensively damaged and there are no lane markings. In our future work, we intend to study how to improve lane detection on unstructured and defected roads. Moreover, we want to develop a robotic vehicle and implement our model to test the robustness of our model in real-life scenarios by measuring the performance of the robot.

## Figures and Tables

**Table 1:** Model Performance Comparison with other State-of-the-art models.

Model	Accuracy (%)	Dice Coefficient (%)	IoU (%)	Dice Loss(%)	Number of Parameters (Million)	File Size (Mb)
PSPNet	95.89	95.46	94.82	5.01	0.33	4.08
U-net	96.27	98.02	96.98	1.98	1.94	22.97
FCN	96.30	98.13	97.19	1.87	1.37	16.35
Ours	96.31	98.18	97.33	1.82	0.26	1.88

## Data Availability

Not applicable.
